# Solitary fibrous tumor of the bladder: Diagnostic challenges and surgical management in an elderly male, a case report and literature review

**DOI:** 10.1016/j.ijscr.2025.111053

**Published:** 2025-02-12

**Authors:** Ahmed Ibrahimi, Reda Tariqi, Mohammed Ali Mikou, Imad Boualaoui, Hachem El Sayegh, Yassine Nouini

**Affiliations:** Department of Urologic Surgery “A” Ibn Sina University Hospital, Mohammed V University, Rabat, Morocco

**Keywords:** Solitary fibrous tumor, Bladder tumor, Renal failure, Surgical resection

## Abstract

Introduction: Solitary fibrous tumors (SFTs) of the bladder are rare mesenchymal neoplasms that mimic other bladder tumors, requiring a multidisciplinary approach for diagnosis and management.

**Case presentation:**

An 81-year-old male with a 30-pack-year smoking history presented with clotting hematuria and acute renal failure. Imaging revealed a large bladder mass causing bilateral ureteral obstruction. Transurethral resection and immunohistochemical analysis confirmed an SFT. The patient underwent cystoprostatectomy with Bricker diversion, achieving full recovery.

**Discussion:**

Bladder SFTs are often misdiagnosed as other spindle cell tumors. Histopathology and CD34 immunopositivity are key for diagnosis. Although typically benign, recurrence and metastasis require long-term follow-up.

**Conclusion:**

This case underscores the rarity of bladder SFTs and highlights the importance of accurate diagnosis and complete surgical excision for optimal outcomes.

## Highlights

• Rare presentation: Solitary fibrous tumours (SFTs) of the bladder are rare and mimic other neoplasms, posing diagnostic challenges.
• Case description: An 81-year-old smoker had total haematuria and renal failure due to a large bladder SFT causing bilateral ureteral obstruction.
• Histopathological diagnosis: Histopathology and CD34-positive immunohistochemistry confirmed the solitary fibrous tumour diagnosis.
• Surgical management: Cystoprostatectomy with Bricker diversion was performed due to extensive tumour involvement and urinary obstruction.
• Long-term follow-up: Despite a favourable prognosis, regular follow-up is needed to detect recurrence or metastasis.

## 1 Introduction

Solitary fibrous tumors (SFTs) are rare mesenchymal neoplasms originating from CD34-positive dendritic mesenchymal cells that most commonly arise in the pleura but can also occur virtually anywhere throughout the body [1], including the urinary bladder [2]. First described in the bladder in 1997, bladder SFTs remain rare, with only a handful of reported cases [3]. Their clinical, radiological, and histological overlap with other bladder neoplasms, such as leiomyomas, sarcomas, and sarcomatoid carcinomas, makes diagnosis challenging. Bladder SFTs may present with nonspecific symptoms such as hematuria, urinary retention, or pelvic pain. Given their rarity and variable presentation, a multidisciplinary approach involving advanced imaging, histopathology, and immunohistochemistry is essential for accurate diagnosis [4].

This report describes a rare case of bladder SFT in an elderly male presenting with total clotting hematuria and acute renal failure. The case highlights the diagnostic and therapeutic challenges of managing bladder SFTs and underscores the importance of comprehensive evaluation and long-term follow-up.

## 2 Case presentation

**Fig. 1:**
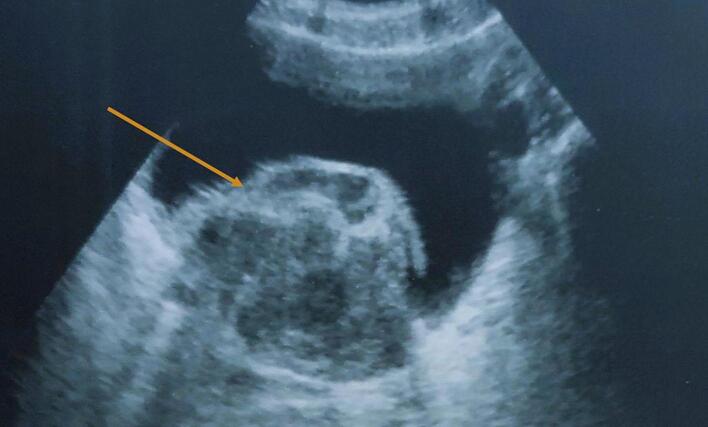
Image of a transverse ultrasound scan of the bladder revealing a circumferential, endoluminal and proliferative bladder wall thickening measuring 38.

An 81-year-old male, a chronic smoker with a 30-pack-year history, was admitted with total clotting hematuria and acute renal failure. Ultrasound revealed circumferential, endoluminal bladder wall thickening measuring 38 mm, along with bilateral pyelocaliceal dilation (Fig. 1).

Laboratory tests showed anemia (hemoglobin: 10 g/dL) and renal insufficiency with a serum creatinine level of 8.8 mg/dL and blood urea nitrogen (BUN) of 267 mg/dL.

Transurethral resection of the bladder (TURB) revealed a large, solid, non-papillary mass arising from the trigone and posterior wall, infiltrating the ureteral orifices and occupying most of the bladder lumen. Bilateral nephrostomy drainage was performed. Histopathological analysis demonstrated spindle cell proliferation, necessitating immunohistochemical evaluation, which confirmed an SFT. The tumor was strongly positive for CD34 and negative for AE1/AE3, P63, CD31, AML, H-caldesmon, CD117, DOG1, S100, desmin, and ALK.

**Fig. 2:**
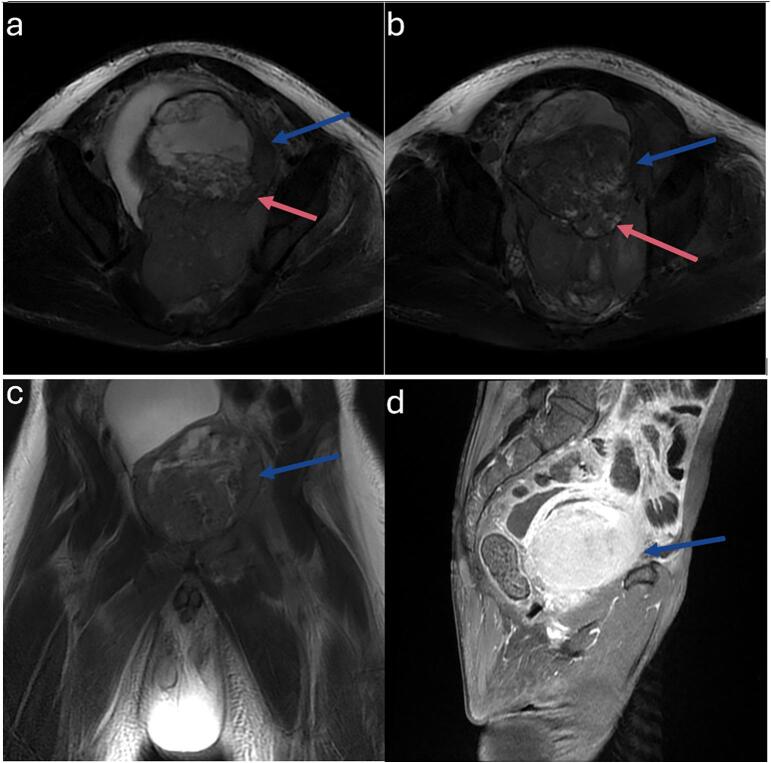
a & b) Axial T2; c) Coronal T2; d) Sagittal T1: MRI scan showed an irregular parietal thickening of the bladder in isosignal T2, T1 and hypersignal on diffusion sequences, clearly enhanced by contrast medium, developed at the expense of the bladder trigone and the posterior wall of the bladder and budded endoluminally creating a mass filling almost the entire bladder lumen. It measures 81 ∗ 78 ∗ 105 mm (blue arrow). The process protruded beyond the posterior bladder wall, breaching the latter and invading the left seminal vesicle (red arrow).

A thoraco-abdominopelvic CT scan showed an 86 × 96 × 115 mm endoluminal bladder tumor with no evidence of metastasis. MRI revealed irregular bladder wall thickening with isointense T1 and T2 signals, hyperintense diffusion sequences, and significant enhancement with contrast. The tumor originated from the trigone and posterior wall, filling nearly the entire bladder lumen and infiltrating both ureteral orifices, causing upstream ureteral dilation. It measured 81 × 78 × 105 mm and extended beyond the posterior bladder wall, invading the left seminal vesicle (Fig. 2).

**Fig. 3:**
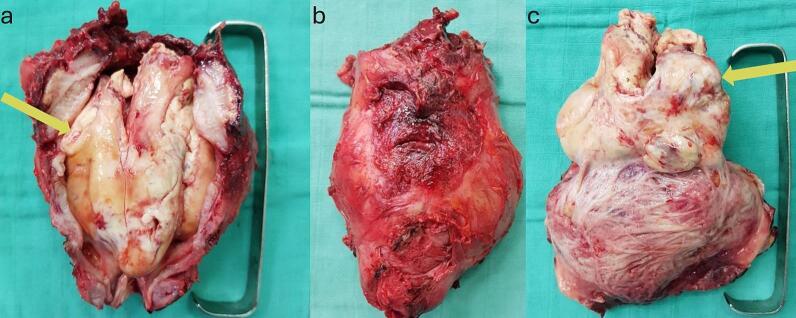
Images of the operative specimen, showing the macroscopic appearance of the tumor, ovoid shaped, well demarcated and encapsulated mass (yellow arrow). a) Rear view; b) side view; b) front view. (For interpretation of the references to color in this figure legend, the reader is referred to the web version of this article.)

A multidisciplinary team decided to perform a total cystoprostatectomy with Bricker urinary diversion due to active bleeding and upper urinary tract obstruction leading to renal failure (Fig. 3). The postoperative course was uneventful, and the patient was discharged on postoperative day 7.

Histopathological analysis of the surgical specimen confirmed an SFT infiltrating the bladder wall up to the perivesical fat, with no involvement of the prostate or seminal vesicles. Urethral and ureteral margins were tumor-free. Clinical and radiological follow-up over two years showed no evidence of recurrence.

## 3 Discussion

Bladder SFTs are exceptionally rare mesenchymal neoplasms [1], constituting a small subset of extrapleural SFTs. Initially described in the pleura, SFTs have since been reported in various anatomical locations, including the bladder. Their rarity and histological overlap with other spindle-cell tumors make diagnosis challenging [2].

This case illustrates a typical but unique presentation of bladder SFT in an elderly male, manifesting as hematuria with acute renal failure due to bilateral ureteral obstruction. While bladder SFTs have been reported in limited cases [4], they remain difficult to diagnose without histopathological and immunohistochemical confirmation.

Comparing this case with previously reported bladder SFTs, our patient presented with significant renal impairment due to bilateral ureteral obstruction, a feature that is not commonly described. Most reported bladder SFTs manifest as localized masses without severe upper urinary tract involvement, making this case particularly notable. Furthermore, the tumor size (81 × 78 × 105 mm) was among the larger bladder SFTs described in the literature. [5,6]

Histologically, bladder SFTs present as well-demarcated intraluminal masses with spindle-cell proliferation. Differential diagnoses include leiomyomas, leiomyosarcomas, sarcomatoid carcinoma, and hemangiopericytomas [7]. Immunohistochemical markers are essential for differentiation, with CD34 being a highly sensitive marker for SFTs. The tumor in this case was negative for markers such as CK, CD31, and S100, excluding other differential diagnoses [8]. The tumor exhibited characteristic imaging features on CT and MRI, including well-defined, heterogeneously enhancing masses with significant endoluminal growth. MRI findings of T1 and T2 isosignals with contrast enhancement and ADC restriction correlated well with histopathological features, emphasizing the role of advanced imaging in preoperative planning [9,10].

Surgical excision with negative margins remains the cornerstone of treatment [11]. In this case, cystoprostatectomy with Bricker diversion was performed due to extensive tumor invasion and its impact on renal function. Postoperative histopathology confirmed a benign SFT without evidence of malignancy or metastasis.

Although most bladder SFTs are benign, recurrence and metastasis have been reported in 10–30 % of cases [2]. Risk factors for malignancy include hypercellularity, high mitotic rate, nuclear pleomorphism, and necrosis [12]. These features were absent in this case, suggesting that complete surgical excision was effective. The patient remains disease-free at two years, underscoring the importance of long-term follow-up with periodic imaging and clinical evaluation. While adjuvant therapy is not routinely recommended, surveillance is essential due to the potential for late recurrence or malignant transformation.

This case emphasizes the need to consider SFTs in the differential diagnosis of bladder spindle-cell neoplasms. Accurate diagnosis and management require recognition of its distinct histological and immunohistochemical profile. Although prognosis is generally favorable for benign bladder SFTs, long-term surveillance is necessary to detect potential recurrences.

## 4 Conclusion

This case highlights the rarity and diagnostic challenges of bladder solitary fibrous tumors (SFTs), despite their typically benign nature. The patient presented with acute renal failure due to bilateral ureteral obstruction caused by the tumor, requiring a combination of histopathological, immunohistochemical, and advanced imaging techniques for accurate diagnosis. Surgical resection with negative margins proved effective, though long-term follow-up remains crucial due to the risk of recurrence. This case underscores the importance of considering SFTs in the differential diagnosis of bladder neoplasms and the necessity of a multidisciplinary approach to optimize patient outcomes.

This work has been reported in line with the SCARE criteria [13].

## Author contribution

‐ Ahmed Ibrahimi, Assistant professor contributed in the correction the case report.
‐ Reda Tariqi, Urology doctor: has contributed in the writing and correction the case report.
‐ Mohammed Ali Mikou, Urology Resident: has contributed in the writing and Data collection.
‐ Imad Boualaoui Assistant professor contributed in the correction the case report.
‐ Hachem El Sayegh,Urology Professor: has contributed in the correction the case report
‐ Yassine Nouini, Urology Professor: has contributed in the correction the case report.

## Informed consent

Written informed consent was obtained from the patient for publication and any accompanying images. A copy of the written consent is available for review by the Editor-in-Chief of this journal on request.

## Ethical approval

Ethical approval is not required by our institution (Centre Hospitalier Universitaire Ibn Sina).

Our case report is exempt from ethical approval as it involves a retrospective and observational analysis, maintains strict patient anonymity and confidentiality, adheres to ethical guidelines, aligns with our institution's policy, and has been conducted with the full and informed consent of the patient involved, which does not mandate approval for such studies.

## Guarantor

Dr Ibrahimi Ahmed.

Dr Tariqi Reda.

## Research registration number

Not applicable.

## Funding

No financial support or grant funding was received.

## Conflict of interest statement

The authors declare no competing interests.

## References

[1] Li, Cao RNa; Yang, Jianyang MMb; Chen, Hongli MMc; Yang, Lie MDa,d,*. A giant solitary fibrous tumor of the abdominal pelvic cavity: a case report and literature review. Medicine 103(32):p e39270, August 09, 2024. 10.1097/MD.0000000000039270. PMC11315500 39121255

[2] Tissue Soft, Tumours Bone (2020).

[3] Bainbridge T.C., Singh R.R., Mentzelt, Katenkampd (1997). Soli tary fibrous tumor of urinary bladder: report of two cases. Hum. Pathol..

[4] Ong Katherine, Singh Shivani, Swarbrick Nicole, Hayne Dickon (2023). Solitary fibrous tumour of the urinary bladder – a rare and potentially malignant neoplasm. Urol. Case Rep..

[5] Li, Tian Yu MM^a^; Zhang, Bo MB^b,⁎^; Zhang, Ji MD^b^. A case report and literature review on primary solitary fibrous tumor of the bladder. Medicine 102(19):p e33708, May 12, 2023. 10.1097/MD.0000000000033708. PMC10174389 37171342

[6] Limsirilak T. (2023). Solitary fibrous tumor of the urinary bladder. Health Sci. Clin. Res..

[7] Tanaka E.Y., Buonfiglio V.B., Manzano J.P., Filippi R.Z., Sadi M.V. (2016). Two cases of solitary fibrous tumor involving urinary bladder and a review of the literature. Case Rep. Urol..

[8] Geramizadeh B., Marzban M., Churg A. (2016). Role of immunohistochemistry in the diagnosis of solitary fibrous tumor, a review. Iran. J. Pathol..

[9] Wong-You-Cheong J.J., Woodward P.J., Manning M.A., Sesterhenn I.A. (2006). From the archives of the AFIP: neoplasms of the urinary bladder: radiologic-pathologic correlation. Radiographics.

[10] Vargas F., Gandhi Darshan, Bajaj Divyansh, Serhal Muhamad, Erazo MD Ibeth S., Singh Jagmeet (December 2021). Solitary fibrous tumor of the urinary bladder: an unusual case report with literature review. Radiol. Case Rep..

[11] Park S.B., Park Y.S., Kim J.K., Kim M.H., Oh Y.T., Kim K.A. (2011). Solitary fibrous tumor of the genitourinary tract. AJR Am. J. Roentgenol..

[12] Demicco E., Wagner M., Maki R. (2017). Risk assessment in solitary fibrous tumours: validation and refinement of a risk stratification model. Mod. Pathol..

[13] Sohrabi C., Mathew G., Maria N., Kerwan A., Franchi T., Agha R.A. (2023). The SCARE 2023 guideline: updating consensus Surgical CAse REport (SCARE) guidelines. Int. J. Surg. Lond. Engl..

